# Validity and reliability of an unstable board for dynamic balance assessment in young adults

**DOI:** 10.1371/journal.pone.0280057

**Published:** 2023-01-06

**Authors:** Alex Rizzato, Erica Gobbi, Antonio Paoli, Giuseppe Marcolin

**Affiliations:** 1 Department of Biomedical Sciences, University of Padova, Padova, Italy; 2 Department of Biomolecular Sciences, University of Urbino Carlo Bo, Urbino, Italy; University of Cassino e Lazio Meridionale, ITALY

## Abstract

Scientific literature is giving greater importance to dynamic balance in fall prevention. Recently, the validity and reliability of the most employed functional tests for dynamic balance assessment has been investigated. Although these functional tests are practical and require minimal equipment, they are inherently subjective, as most do not use instrumented measurement data in the scoring process. Therefore, this study aimed to assess the validity and reliability of an instrumented unstable board for dynamic balance objective assessment in young adults through double-leg standing trials. A test-retest design was outlined with the unstable board positioned over a force platform to collect objective Center of Pressure (CoP) related and kinematic parameters. Fifteen young adults participated in two evaluation sessions (7-day apart) that comprised ten trials per two dynamic conditions (anterior-posterior and medio-lateral oscillations) aiming to maintain the board parallel to the ground. Pearson’s correlation coefficient (r) was employed to assess the validity of the kinematic parameters with those derived from the CoP. The test-retest reliability was investigated through Intraclass Correlation Coefficient (ICC), Standard Error of the measurement, Minimal Detectable Change, and Bland-Altman plots. Statistically significant correlations between the CoP and kinematic parameters were found, with r values ranging from 0.66 to 0.95. Good to excellent intrasession (0.89≤ICCs≤0.95) and intersession (0.66≤ICCs≤0.95) ICCs were found for the kinematics parameters. The Bland-Altman plots showed no significant systematic bias. The kinematics parameters derived from the unstable board resulted valid and reliable. The small size of the board makes it a suitable tool for the on-site dynamic balance assessment and a complement of computerized dynamic posturography.

## Introduction

In humans, the habitual stance depends on maintaining vertical balance, as in no other species on earth [1]. Indeed, a set of structures [2] jointly work to control body segments against gravity and to maintain the center of pressure (CoP) within the base of support [3]. An efficient postural balance control prevents falls [4] and injuries [5] but also contributes to optimizing sports performance [6, 7]. Thus, the accuracy in quantifying postural balance is crucial and needs valid and reliable assessment techniques. To this extent, force platforms are the most widely used devices in assessing postural function [8]. However, considering the high cost of force platforms, some researchers focused on more inexpensive systems, showing, for instance, that a game-oriented platform could be suitable for static balance assessment [9]. Scientific literature is giving greater importance to dynamic balance management and training [10, 11], referring to the ability to react efficiently to external mechanical stimuli [12] or even to virtual reality scenarios [13, 14]. Indeed, dynamic balance likely demands different biomechanical and neuromuscular control strategies [15] compared to static balance. Consequently, static and dynamic balance are required to be better assessed complementarily, being independent of each other [16]. To this extent, several studies have investigated the validity and reliability of the most employed functional tests for dynamic balance assessment. Findings highlighted good validity and reliability among older, frailer adults but showed ceiling effects when applied in high-functioning older adults [17–23]. Indeed, these tests resulted in undemanding for healthy people and even more for athletes, failing to elicit postural stability deficiencies and discriminate the performance level [24]. Further, despite functional tests are practical, inexpensive, and require minimal equipment, they are not associated with objective CoP-related parameters derived from force platforms. About that, Petrò and colleagues highlighted the spread of dynamic balance tests and proposed a categorization of the existing objective methods [25], finding significant employment of unstable boards. An unstable board could provide constant instability pivoting around a fulcrum due to its center of mass located above the pivot [26]. These devices have been proved to fit with both athletes [27] and balance-affected people [28]. Moreover, their engineering is relatively inexpensive and does not affect their transportability. On this point, Orrell and colleagues instrumented an oscillating wooden platform with a potentiometer to transform the linear displacement of the board into angular degrees out from the horizontal [29]. Similarly, Marcolin and colleagues employed a 3D inertial measurement unit (IMU) to calculate dynamic balance parameters from a wooden board oscillating around its pivot axis [27]. Although these approaches are promising and more challenging than most dynamic functional tests, they lacked to consider the reliability of their outputs compared to the CoP-related parameters derived from force platforms. Fusco and colleagues first tried to determine the reliability and validity of a computerized wobble board [30]. Although they found acceptable levels of error and low minimal detectable changes, wobble board indexes were correlated with indexes derived from a functional test (i.e., the Y Balance Test), again, without considering CoP-related parameters. Recently, Rougier and colleagues studied dynamic balance control positioning a double seesaw over two force platforms allowing pitch motions and collected the CoP displacements under each foot in the standing position [31]. Although this is a reliable method for studying postural strategies under dynamic conditions, the essential requirement of force platforms makes this approach expensive and hardly applicable in the field. Therefore, the aims of the present study were: (i) to determine the validity of the kinematic parameters obtained from an instrumented unstable board compared to the CoP-related parameters derived from a force platform; (ii) to evaluate the reliability of a double-leg stance test on an instrumented unstable board among healthy young adults. We hypothesized that the kinematic parameters of the instrumented wooden board could be reliable and valid field measures of the dynamic balance performance.

## Materials and methods

### Subjects

We performed an a-priori sample size calculation based on the formulas proposed for estimating ICC in biomedical reliability studies [32, 33]. Specifically, by setting a minimum acceptable reliability ICC at 0.65, with a significance level at 0.05, power at 80%, and two repetitions (i.e., inter-sessions reliability), 15 subjects represented the required sample size. This was the worst-case scenario, as by setting a minimum acceptable reliability ICC at 0.65, with a significance level at 0.05, power at 80%, and 10 repetitions (i.e., intra-session reliability), the required sample size would be 3 subjects. Consequently, we enrolled 15 healthy young subjects (M = 9, F = 6; mean ± SD: 23.13 ± 0.99 years; 71.67 ± 12.37 kg; 1.77 ± 0.082 m) with no history of (i) orthopedic injuries in the last year, (ii) neurological diseases, and (iii) sight, hearing, or vestibular disorders. All subjects were physically active and involved in at least one recreational sport activity twice per week.

### Experimental design

The experimental protocol received approval from the Human Ethical Committee of the Department of Biomedical Science of the University of Padova (n° HEC-DSB/08-18). All methods were performed in accordance with the Declaration of Helsinki. Subjects were informed about the methods and aims of the study, gave their written informed consent, and were free to renounce the study at any stage. The week before testing, researchers organized a session explaining in detail the scheduled program letting the subjects familiarize themselves with the instrumentation.

**Fig 1:**
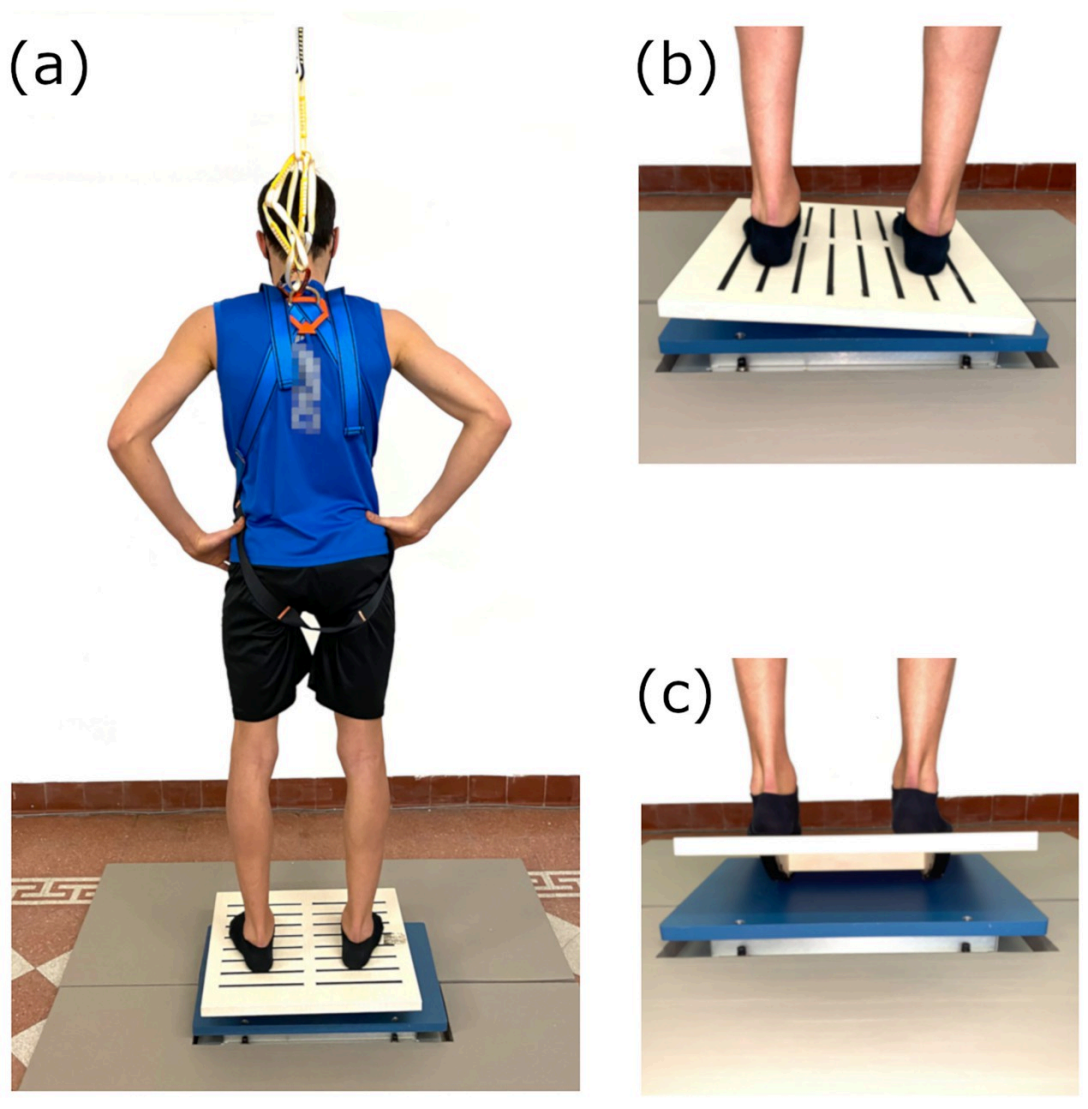
Experimental setup. Subject secured to a harness standing on the unstable board placed over the force platform (a); detail of the force platform and the unstable board during medio-lateral (b) and anterior-posterior (c) dynamic tests.

A cross-sectional test-retest design was outlined to assess postural balance control under two different dynamic conditions. For all subjects, retest trials (DAY 2) were performed a week after the test trials (DAY 1). Subjects were instructed to stand on an unstable square board with parallel feet, according to specific lines drawn on the surface of the board. Subsequently, they were asked to maintain the board parallel to the ground as much as possible without moving the feet from their original position. For the test duration, subjects gazed at a thin green line vertically placed in front of them on a white wall at 80 centimeters, keeping their hands on hips to avoid counterbalance actions. In case of loss of balance, subjects were secured with a harness. The unstable square board was positioned over a force platform (AMTI BP400600, Watertown, MA, USA) to record its oscillations together with the CoP trajectory synchronously (Fig 1). For this purpose, two reflective markers were placed at the vertices of the unstable board and their three-dimensional trajectories were recorded by a 6-camera optoelectronic system (OptiTrack—Natural Point Inc.).

Trials were performed in both dynamic anterior-posterior (AP) and medio-lateral (ML) directions, separately (Fig 1). During the AP condition, the subjects’ sagittal axis was parallel to the rotational axis of the unstable board. In contrast, during the ML condition, the subjects’ sagittal axis was perpendicular to the rotational axis. Overall, subjects performed ten trials in both dynamic conditions. The duration was set to 30 seconds for all trials, according to Scoppa and colleagues’ guidelines on stabilometric tests over force platforms [34]. The rest between the trials was set to 60 seconds.

### Measurements

**Fig 2:**
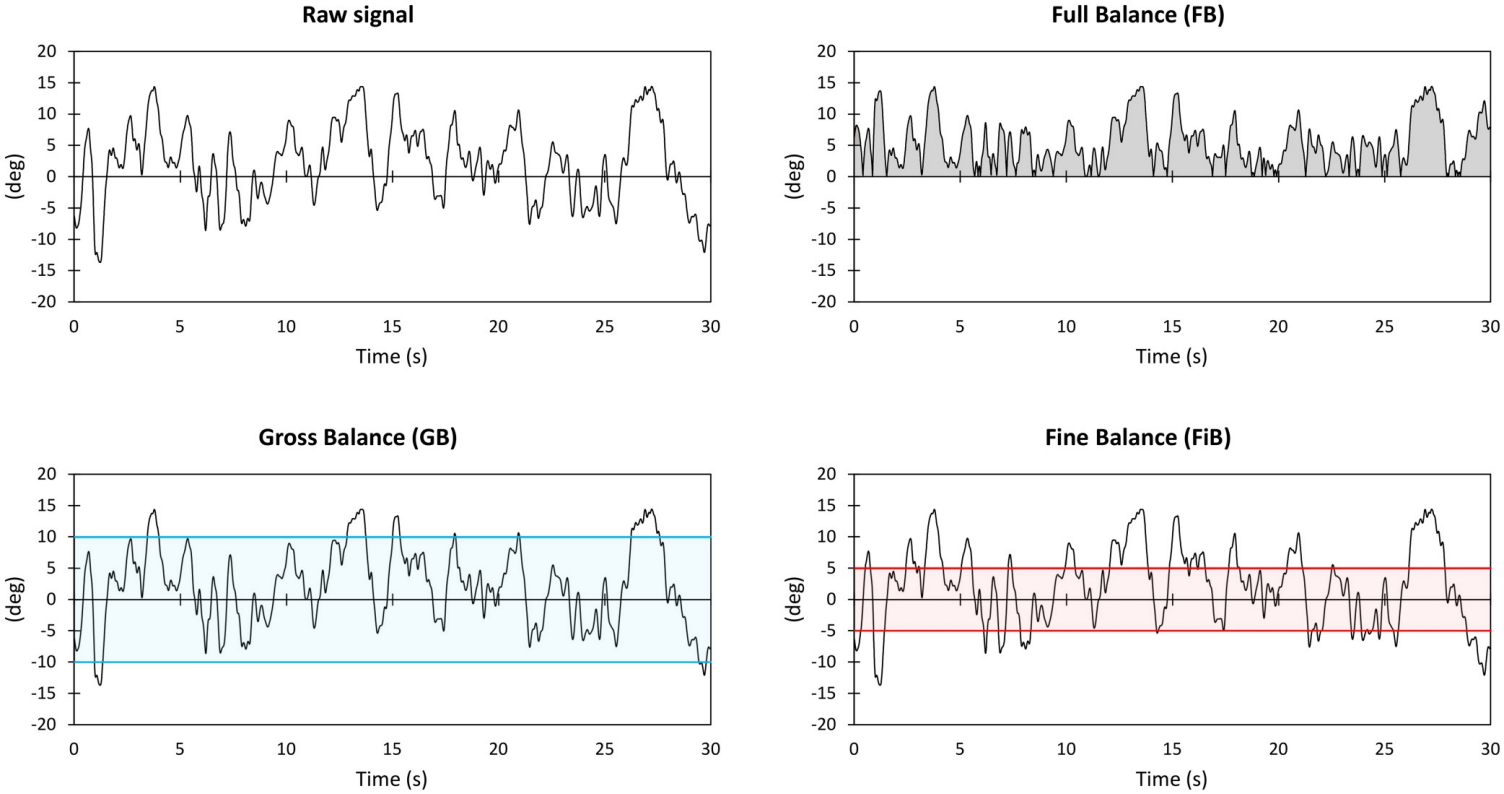
Graphical representation of the kinematic parameters (i.e., Full Balance, Gross Balance, and Fine Balance) in a representative AP trial. Full balance is the area below the rectified raw signal and is represented in grey. Gross Balance is the total time the raw signal stays between +10deg and -10deg, represented in blue. Fine Balance is the total time the raw signal stays between +5deg and -5deg, represented in red.

The CoP trajectory was recorded through the computerized force platform at a sampling frequency of 100 Hz. The platform was zeroed according to the manufacturer’s guidelines before recording each trial. The platform employed in this study has an average CoP accuracy of less than 0.2 mm, crosstalk values ± 0.05% of the applied load, and a measurement accuracy typically ± 0.1% of the applied load (minimum applied load of 22.6 kg). The CoP signal was analyzed with the software Balance Clinic 1.4.2 (AMTI, Watertown, MA, USA). In each condition, Area95 (the area of the 95th percentile ellipse measured in cm^2^) and Unit Path (the path length per unit time, i.e., the average CoP velocity measured in cm/s) were considered as resulting outputs. The kinematic data were recorded at 100 Hz for consistency with the kinetic data. The two reflective markers on the edge of the unstable board allowed calculating the rotation angle of the square board: when the markers were parallel to the floor, the angle was 0 deg. Positive and negative angle values were measured when the unstable board rotated clockwise or counterclockwise. Three parameters (Fig 2) were calculated to assess the dynamic balance performance: the integral of the curve considered an index of the overall postural performance (Full Balance, FB); the time spent between +5 deg and -5 deg considered an index of fine-tuning balance adjustments (Fine Balance, FiB); the time spent between +10 deg and -10 deg considered an index of gross-tuning balance adjustments (Gross Balance, GB). FB was considered the primary measure in the validity and reliability assessment, while FiB and GB as the secondary ones. We employed a motion capture system because it represents the gold standard in kinematic analysis. Results were analyzed for both AP and ML conditions; a mean score between AP and ML conditions (i.e., Total Dynamic Score, TDS) was also considered.

### Statistical analyses

The mean values and standard deviation (SD) were calculated for all variables. Pearson’s correlation coefficient was employed to assess the convergent validity of the measures, correlating the CoP-related parameters (i.e., Area95 and Unit Path) with kinematic parameters (i.e., FB, FiB, and GB). The strength of the correlations was interpreted following Cohen and colleagues [35] as absent to little (<0.25), fair (0.25–0.49), moderate (0.5–0.74), and very good to excellent (> 0.75). The significance level was set at p < 0.05.

The test-retest reliability of the kinematic parameters was investigated through Intraclass Correlation Coefficient (ICC) and the relative 95% confidence interval (95% CI), Standard Error of the measurement (SEm), Minimal Detectable Change (MD_95_), and Bland-Altman plots. ICC, which represents both degrees of correlation and agreement between measurements, was employed to assess the intrasession and intersession reliability for the FB, FiB, and GB measurements. A 2-way approach was used to calculate ICCs, being the trials the substitutes for raters. According to this model, our results only represented the reliability of the specific measurements of the experiment. Intersession ICC [3,1] estimated correlations between DAY 1 and DAY 2 balance measurements, and intrasession ICC [3, *k*] estimated correlations averaged between the *k* measurements, where in our study *k* = 10. ICC coefficients were interpreted as poor (0.00–0.39), fair (0.40–0.59), good (0.60–0.74), and excellent (0.75–1.00) [36].

The SEm is the standard error in estimating observed scores from true scores [37]. It was compounded for intersession reliability through the following formula:

SEm=SDd/√2

where SD*d* represents the SD of the differences between test and retest scores (*d*). Further, the SEm was used to determine the minimum difference (MD_95_) according to the following formula:

$${\text{MD}}_{95}=\text{SEm}\cdot 1.96\cdot \surd 2.$$

More precisely, for all people whose differences on repeated testing were at least greater than or equal to the MD, 95% of them would reflect real differences [37]. The lower the SEm and MD_95_, the higher the intersession reliability.

Finally, Bland-Altman plots visually showed the level of agreement and the 95% limits of agreement (LoA_95_) by plotting the paired differences vs. the pair-wise means. LoA_95_ estimated the interval within which a proportion of the differences between measurements lies with 95% of certainty [38]. LoA_95_ was calculated as follows:

$${\text{LoA}}_{95}=\text{mean}\pm 2\;\text{SD}$$

Thus, a real change in the subject’s performance occurs when the difference between the two measures falls outside the LoA_95_. All analyses were performed using Statistical Package for Social Sciences (SPSS) version 27 (IBM, Armonk, New York, USA).

## Results

Table 1 shows for each subject the mean scores and standard deviations (SD) averaged across the ten trials and the group means (GM) of both the CoP-related parameters.

**Table 1:** Mean values, group means (GM), and standard deviations (SDs) of the CoP-related parameters (Area95 and Unit Path) during the unstable board test (DAY1) performances.

	DAY 1					
	Area95 (cm^2^)			Unit Path (cm/s)		
	AP	ML	Mean	AP	ML	Mean
S1	7.87 ± 2.60	17.56 ± 6.42	12.72 ± 4.06	4.61 ± 0.34	5.62 ± 0.63	5.12 ± 0.43
S2	18.53 ± 5.62	25.67 ± 10.90	22.10 ± 7.10	8.76 ± 0.92	9.44 ± 1.26	9.11 ± 0.92
S3	14.96 ± 2.60	14.83 ± 3.34	14.90 ± 2.46	6.83 ± 0.72	7.33 ± 0.81	7.08 ± 0.70
S4	23.01 ± 3.45	50.08 ± 11.49	36.55 ± 6.40	7.61 ± 1.05	10.69 ± 2.23	9.16 ± 1.53
S5	20.93 ± 5.22	30.44 ± 10.04	25.69 ± 4.83	8.92 ± 1.21	9.68 ± 1.06	9.30 ± 0.66
S6	18.21 ± 4.74	25.56 ± 8.38	21.89 ± 3.99	6.02 ± 0.82	7.18 ± 1.12	6.61 ± 0.58
S7	7.141 ± 1.94	20.35 ± 9.32	13.75 ± 4.25	5.98 ± 0.71	7.18 ± 1.16	6.58 ± 0.64
S8	13.98 ± 4.53	28.70 ± 5.29	21.34 ± 3.47	5.42 ± 0.35	7.56 ± 0.73	6.49 ± 0.43
S9	11.69 ± 3.41	44.83 ± 15.81	28.27 ± 7.38	7.25 ± 0.85	11.27 ± 1.10	9.27 ± 0.63
S10	32.51 ± 10.21	29.51 ± 7.21	31.01 ± 6.58	8.69 ± 0.97	8.42 ± 0.92	8.56 ± 0.69
S11	14.35 ± 3.68	30.32 ± 6.21	22.34 ± 2.98	6.86 ± 0.64	9.00 ± 0.88	7.93 ± 0.58
S12	18.00 ± 4.17	45.84 ± 10.41	31.92 ± 5.67	7.18 ± 0.77	11.24 ± 1.29	9.22 ± 0.69
S13	13.45 ± 4.52	37.32 ± 9.14	25.39 ± 5.03	7.44 ± 0.93	10.25 ± 1.39	8.85 ± 0.70
S14	17.92 ± 4.72	22.38 ± 4.99	20.16 ± 3.07	8.27 ± 0.91	7.88 ± 1.00	8.08 ± 0.63
S15	15.07 ± 4.21	91.75 ± 38.30	53.41 ± 20.82	6.94 ± 1.00	17.30 ± 4.42	12.12 ± 2.67
***GM ± SD***	***16*.*51 ± 6*.*20***	***34*.*34 ± 18*.*96***	***25*.*43 ± 10*.*26***	***7*.*12 ± 1*.*25***	***9*.*34 ± 2*.*76***	***8*.*23 ± 1*.*69***

Tables 2 and 3 report kinematic results recorded in the two sessions and referred to the board oscillations.

**Table 2:** Mean values, group means (GM), and standard deviations (SDs) of the kinematic parameters (Full balance, Fine Balance, and Gross Balance) during the unstable board test (DAY 1) and retest (DAY 2) performances.

	**DAY 1**					
	**Full Balance (deg∙s)**		**Fine Balance (s)**		**Gross Balance (s)**	
	**AP**	**ML**	**AP**	**ML**	**AP**	**ML**
S1	154.73 ± 23.28	138.56 ± 21.23	16.59 ± 2.88	18.59 ± 2.05	26.05 ± 1.90	26.77 ± 1.50
S2	181.56 ± 26.04	161.17 ± 21.93	14.62 ± 2.30	16.12 ± 2.45	23.67 ± 2.47	25.36 ± 1.83
S3	161.08 ± 19.15	118.46 ± 18.63	15.72 ± 2.07	20.69 ± 2.23	25.80 ± 1.74	28.62 ± 1.19
S4	203.39 ± 19.79	202.22 ± 16.55	12.46 ± 2.18	12.45 ± 1.65	21.90 ± 1.53	22.11 ± 1.81
S5	157.36 ± 19.54	148.69 ± 21.08	16.26 ± 2.04	17.81 ± 2.78	26.14 ± 2.01	26.55 ± 1.28
S6	181.46 ± 21.04	161.67 ± 24.61	14.30 ± 2.20	15.80 ± 2.20	23.76 ± 1.83	25.67 ± 2.19
S7	118.47 ± 12.99	137.78 ± 28.80	20.82 ± 1.82	18.80 ± 2.98	28.82 ± 1.29	27.10 ± 2.23
S8	157.73 ± 19.83	164.03 ± 20.59	16.71 ± 2.05	16.37 ± 2.30	26.20 ± 1.55	25.07 ± 1.47
S9	142.26 ± 24.79	170.99 ± 17.96	18.27 ± 2.41	15.32 ± 1.73	26.79 ± 2.12	24.49 ± 1.45
S10	194.27 ± 29.33	169.90 ± 22.68	13.33 ± 2.67	15.50 ± 2.59	22.63 ± 2.41	24.61 ± 1.69
S11	151.31 ± 21.21	171.91 ± 14.30	17.38 ± 2.97	15.19 ± 1.15	26.35 ± 1.17	24.51 ± 1.48
S12	167.85 ± 21.28	188.22 ± 30.56	15.44 ± 2.83	14.30 ± 2.80	25.26 ± 1.29	22.80 ± 2.58
S13	168.36 ± 25.49	191.09 ± 23.90	15.36 ± 2.53	13.37 ± 2.18	25.03 ± 2.02	23.07 ± 1.95
S14	183.05 ± 30.78	147.10 ± 24.21	14.11 ± 3.14	17.37 ± 2.89	23.91 ± 2.76	27.18 ± 1.49
S15	210.39 ± 25.00	255.79 ± 30.57	10.81 ± 2.68	8.87 ± 2.13	22.17 ± 1.83	16.96 ± 2.87
***GM ± SD***	***168*.*89 ± 24*.*13***	***168*.*51 ± 32*.*72***	***15*.*48 ± 2*.*43***	***15*.*77 ± 2*.*87***	***24*.*96 ± 1*.*92***	***24*.*73 ± 2*.*80***
	**DAY 2**					
	**Full Balance (deg∙s)**		**Fine Balance (s)**		**Gross Balance (s)**	
	**AP**	**ML**	**AP**	**ML**	**AP**	**ML**
S1	151.02 ± 24.17	140.61 ± 16.11	17.50 ± 3.53	18.14 ± 1.71	26.03 ± 1.40	27.16 ± 1.33
S2	169.39 ± 16.07	135.80 ± 16.86	15.16 ± 1.79	18.96 ± 1.98	25.08 ± 1.56	27.35 ± 1.31
S3	173.50 ± 24.90	140.35 ± 19.35	14.89 ± 2.52	18.73 ± 1.98	24.48 ± 2.17	26.71 ± 1.55
S4	160.51 ± 31.24	154.41 ± 30.42	16.20 ± 2.18	16.05 ± 1.65	25.47 ± 1.53	26.78 ± 1.81
S5	145.70 ± 23.07	137.80 ± 22.19	17.98 ± 2.81	18.55 ± 2.45	26.72 ± 1.59	27.49 ± 1.41
S6	157.35 ± 28.29	123.79 ± 15.06	16.31 ± 3.45	20.49 ± 1.42	26.20 ± 1.86	27.91 ± 1.19
S7	105.54 ± 23.15	149.87 ± 19.80	22.48 ± 3.20	18.30 ± 2.05	29.23 ± 1.38	25.89 ± 1.78
S8	137.61 ± 34.83	118.89 ± 23.18	19.86 ± 3.98	20.79 ± 2.96	26.33 ± 2.53	28.87 ± 1.19
S9	141.65 ± 32.35	140.91 ± 17.74	17.75 ± 3.68	18.18 ± 1.74	27.15 ± 2.23	27.23 ± 1.73
S10	163.22 ± 20.90	142.69 ± 21.75	15.81 ± 2.49	18.22 ± 2.47	25.47 ± 1.70	26.89 ± 1.79
S11	147.39 ± 25.88	133.33 ± 18.30	17.41 ± 2.96	19.15 ± 2.00	26.45 ± 1.69	27.34 ± 1.25
S12	190.94 ± 28.34	188.74 ± 25.47	13.66 ± 2.62	13.93 ± 2.37	22.91 ± 2.24	23.28 ± 2.10
S13	163.40 ± 16.76	131.03 ± 18.64	16.35 ± 2.13	19.42 ± 2.00	25.07 ± 1.26	27.57 ± 1.15
S14	158.82 ± 24.12	115.63 ± 20.95	15.77 ± 2.59	21.05 ± 2.75	26.30 ± 1.94	28.97 ± 0.85
S15	201.37 ± 30.07	228.41 ± 22.77	12.86 ± 2.83	10.93 ± 1.63	22.17 ± 2.51	19.32 ± 2.51
***GM ± SD***	***157*.*83 ± 22*.*59***	***145*.*48 ± 28*.*67***	***16*.*42 ± 2*.*61***	***17*.*82 ± 2*.*99***	***25*.*44 ± 1*.*95***	***26*.*28 ± 2*.*66***

**Table 3:** Mean values, group means (GM), and standard deviations (SDs) of the kinematic parameters (Full balance, Fine Balance, and Gross Balance) during the unstable board test (DAY 1) and retest (DAY 2) performances. The Total Dynamic Score (TDS) was calculated for each subject averaging the ten AP and ML trials.

	DAY 1			DAY 2		
	Total Dynamic Score (TDS)			Total Dynamic Score (TDS)		
	Full Balance (deg∙s)	Fine Balance (s)	Gross Balance (s)	Full Balance (deg∙s)	Fine Balance (s)	Gross Balance (s)
S1	146.65 ± 19.99	17.59 ± 2.20	26.42 ± 1.42	145.82 ± 1 5.69	17.82 ± 2.30	26.60 ± 1.01
S2	171.37 ± 21.96	15.38 ± 1.95	24.52 ± 2.03	152.60 ± 10.49	17.06 ± 1.37	26.22 ± 0.85
S3	139.78 ± 15.19	18.21 ± 1.57	27.22 ± 1.23	156.93 ± 15.37	16.81 ± 1.51	25.60 ± 1.32
S4	202.81 ± 13.98	12.46 ± 1.17	22.01 ± 1.46	157.46 ± 14.51	12.46 ± 1.17	22.01 ± 1.46
S5	153.03 ± 14.35	17.04 ± 1.85	26.35 ± 1.16	141.76 ± 16.38	18.27 ± 1.92	27.11 ± 0.89
S6	171.57 ± 15.01	15.06 ± 1.46	24.72 ± 1.18	140.57 ± 12.24	18.40 ± 1.48	27.06 ± 0.87
S7	128.13 ± 13.76	19.82 ± 1.25	27.96 ± 1.01	127.71 ± 14.37	20.40 ± 1.95	27.56 ± 1.20
S8	160.89 ± 13.62	16.55 ± 1.87	25.64 ± 0.77	128.25 ± 26.75	20.33 ± 3.22	27.61 ± 1.56
S9	156.63 ± 13.72	16.80 ± 1.24	25.64 ± 1.27	141.29 ± 14.10	17.97 ± 1.54	27.19 ± 1.04
S10	182.09 ± 15.70	14.42 ± 1.45	23.62 ± 1.49	152.96 ± 18.05	17.02 ± 2.15	26.19 ± 1.27
S11	161.62 ± 13.11	16.29 ± 1.49	25.43 ± 0.95	140.37 ± 17.15	18.28 ± 1.99	26.90 ± 1.12
S12	178.04 ± 18.09	14.88 ± 1.96	24.03 ± 1.24	189.85 ± 25.26	13.80 ± 2.40	23.10 ± 1.93
S13	179.73 ± 16.20	14.37 ± 1.63	24.05 ± 1.35	147.22 ± 11.52	17.89 ± 1.39	26.32 ± 0.70
S14	165.08 ± 18.30	15.75 ± 2.10	25.55 ± 1.32	137.23 ± 10.98	18.42 ± 1.39	27.64 ± 0.87
S15	233.09 ± 25.92	9.85 ± 2.14	19.57 ± 2.23	214.89 ± 18.28	11.90 ± 1.61	20.75 ± 1.69
***GM ± SD***	***168*.*70*** ± ***25*.*63***	***15*.*63*** ± ***2*.*39***	***24*.*85 ± 2*.*09***	***151*.*66 ± 22*.*93***	***17*.*12 ± 2*.*52***	***25*.*86 ± 2*.*15***

The results of the correlational analysis are presented in Table 4. Considering the AP condition, we found statistically significant correlations between the Area95 and Full balance (p < 0.01), Fine balance (p < 0.05), and Gross Balance (p < 0.01), respectively. Conversely, we did not detect any significant correlation between the Unit Path and the kinematic parameters. For what concerns the ML condition, statistically significant correlations occurred between the Area95 and Full balance (p < 0.001), Fine balance (p < 0.001), and Gross Balance (p < 0.001), respectively. Unlike the AP condition, statistically significant correlations were found between the Unit Path and Full balance (p < 0.001), Fine balance (p < 0.001), and Gross Balance (p < 0.001) in the ML condition.

**Table 4:** Pearson correlations between CoP-related parameters (Area95 and Unit path) derived from the force platform and kinematic parameters (Full balance, Fine balance, and Gross balance) obtained from the instrumented balance board.

**AP Oscillations**	Full balance (deg·s)	Fine balance (s)	Gross Balance (s)
Area95 (cm^2^)	r = 0.66**	r = -0.61*	r = -0.69**
Unit Path (cm/s)	r = 0.39	r = -0.36	r = -0.41
**ML Oscillations**	Full Balance (deg·s)	Fine Balance (s)	Gross Balance (s)
Area95 (cm^2^)	r = 0.94***	r = -0.90***	r = -0.95***
Unit Path (cm/s)	r = 0.88***	r = -0.84***	r = -0.89***

AP: antero-posterior; ML: medio-lateral. Statistically significant * (p<0.05), ** (p<0.01), and *** (p<0.001).

Results of the intersession and intrasession reliability analysis are provided in Tables 5 and 6.

**Table 5:** Average intersession [3,1] intraclass correlation coefficient (ICC) and 95% Confidence Intervals (95% CI), Standard Error of the measurement (SEm), and Minimal Detectable Change (MD_95_) calculated during the performances on the unstable board.

		ICC (95% CI) Intersession	SEm Intersession	MD_95_ Intersession
**AP**	Full Balance (deg·s)	0.81 (0.36–0.94)	11.67	32.36
	Fine Balance (s)	0.84 (0.35–0.95)	1.06	2.95
	Gross Balance (s)	0.74 (0.27–0.91)	1.11	3.09
**ML**	Full Balance (deg·s)	0.72 (-0.55–0.91)	16.56	45.91
	Fine Balance (s)	0.66 (-0.12–0.89)	1.63	4.52
	Gross Balance (s)	0.74 (-0.20–0.92)	1.33	3.68
**TDS**	Full Balance (deg·s)	0.75 (0.00–0.92)	12.44	34.50
	Fine Balance (s)	0.80 (0.13–0.94)	1.13	3.13
	Gross Balance (s)	0.85 (0.38–0.95)	0.91	2.52

AP: antero- posterior; ML: medio-lateral; TDS: Total Dynamic Score.

**Table 6:** Average intrasession [3, 10] intraclass correlation coefficient (ICC) and 95% Confidence Intervals (95% CI), Standard Error of the measurement (SEm), and Minimal Detectable Change (MD_95_) calculated during the performances on the unstable board.

		ICC (95% CI) Intrasession	SEm Intrasession	MD_95_ Intrasession
**AP**	Full Balance (deg·s)	0.90 (0.82–0.96)	21.58	59.83
	Fine Balance (s)	0.89 (0.79–0.95)	2.36	6.54
	Gross Balance (s)	0.90 (0.87–0.96)	1.68	4.64
**ML**	Full Balance (deg·s)	0.95 (0.90–0.98)	23.20	64.29
	Fine Balance (s)	0.93 (0.87–0.97)	2.29	6.36
	Gross Balance (s)	0.95 (0.91–0.98)	1.91	5.29
**TDS**	Full Balance (deg·s)	0.95 (0.91–0.98)	16.18	44.85
	Fine Balance (s)	0.94 (0.89–0.98)	1.64	4.53
	Gross Balance (s)	0.95 (0.91–0.98)	1.28	3.54

Intrasession parameters are based on DAY 1 data. AP: antero- posterior; ML: medio-lateral; TDS: Total Dynamic Score.

**Fig 3:**
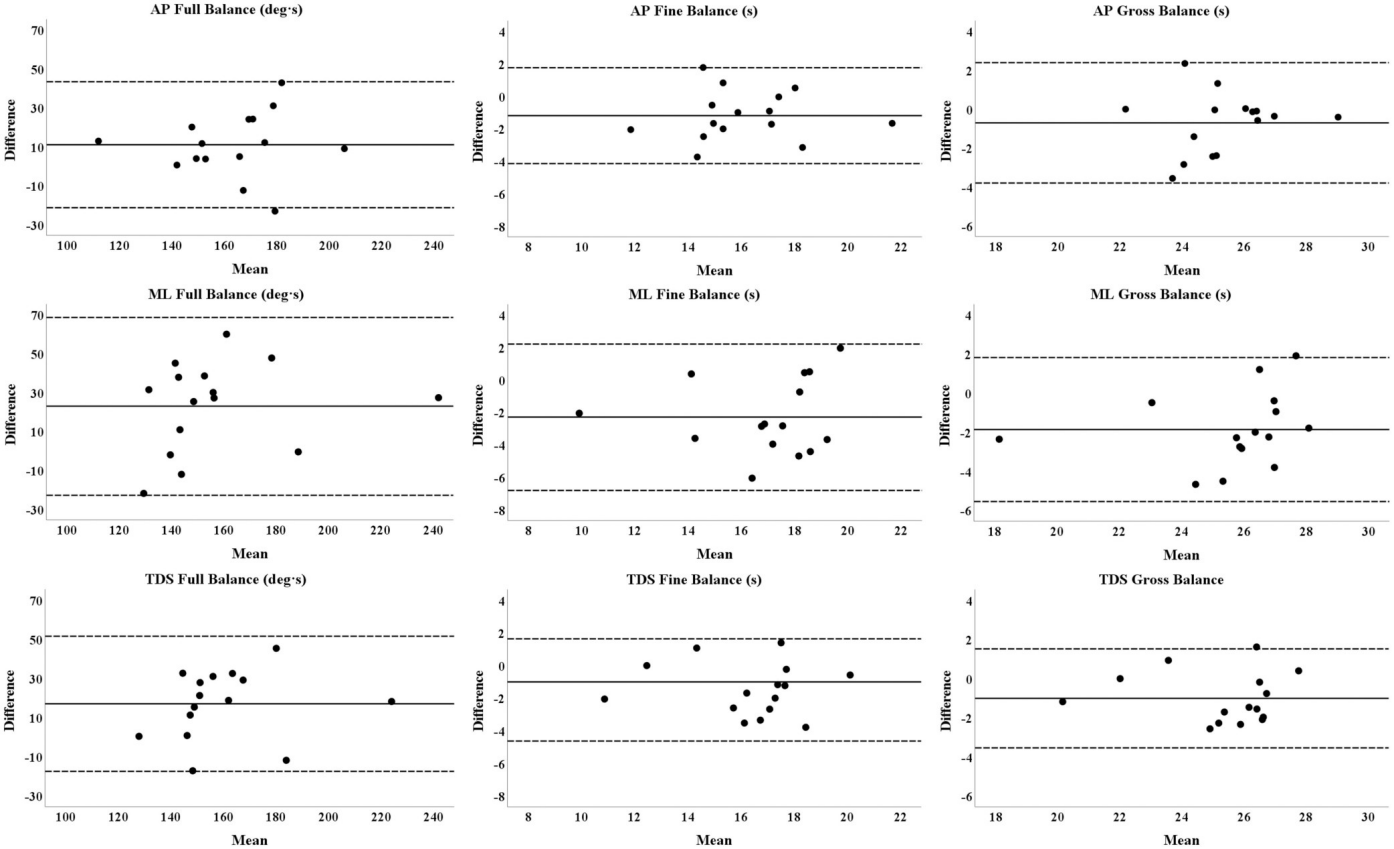
Bland-Altman plot for Full Balance, Gross Balance, and Fine Balance parameters in the anterior-posterior, medio-lateral, and TDS conditions. Differences between test and retest scores are plotted against their means. The black line indicates the average of the differences, whereas the dotted ones represent the 95% limits of agreement.

The kinematic parameters across the AP and ML conditions showed ICC values ranging from good to excellent (0.66 ≤ ICCs ≤ 0.95). SEm and MD_95_ values are reported in Tables 5 and 6. Fig 3 shows, for each kinematic parameter, the Bland-Altman plot representing the differences between test and retest values plotted against their means together with the 95% LoA.

## Discussion

The present study aimed to determine the validity and reliability of a double-leg standing test over an instrumented unstable board. Specifically, the parameters derived from the board oscillations were validated over the CoP-related parameters obtained from the force platform. The kinematic parameters showed excellent correlations in the ML test with both the Area95 and the Unit Path, showing an average *r* equal to 0.93 and 0.87, respectively. In the AP condition, a moderate correlation was detected only with Area95, where the average *r* was equal to 0.65. Since Area95 is considered an index of the overall postural performance [8, 39], we can assume that the kinematic parameters derived from the unstable board motion are valid indexes to quantify the overall dynamic postural performance of an individual objectively. The Unit Path reflects the efficiency of the postural control system characterizing the net neuromuscular activity necessary to maintain the balance [8, 40]. Some authors [41–43] also considered the most sensitive parameter to compare groups of individuals of different ages or with different neurological diseases. Unlike the Area95 results, the moderate correlation detected between the kinematic parameters and the Unit Path in the AP test suggests employing this test with caution if the main goal is to characterize the net neuromuscular activity in maintaining balance. Therefore, findings support using the kinematic parameters obtained from the unstable board (i.e., FB, FiB, and GB) instead of the Area95 derived from the dynamometric platform to objectively quantify the overall dynamic postural performance in AP and ML conditions. Conversely, using FB, FiB, and GB resulted in valid substitutes of the Unit Path derived from the dynamometric platform to assess the dynamic postural balance efficiency only in the ML test. The FB parameter, both in AP and ML, was calculated as the integral of the time-angle curve referred to the unstable board oscillations. Taking as reference that ideally the best performance occurs with the unstable board parallel to the ground for the whole test (i.e., the angle of the board and consequently FB equal to zero), the smaller the FB value, the better the balance performance. The FB could be considered an index of the overall balance performance, and comparable to the Area95 calculated from the COP trajectory in the static stabilometric test. The FiB and GB parameters represent the fine and gross postural balance adjustments, giving additional insight into the amount of time the unstable board lays near the parallel-to-the-ground position.

Given the validity of the kinematic parameters compared to the CoP-related indexes, the second step of the present study was to investigate their reliability. Notably, as presented in Table 6, FB, FiB, and GB showed excellent reliability, with ICC intrasession values ranging from 0.89 to 0.95. Even better, the TDS showed ICC values higher than 0.94. Similarly (Table 5), intersession ICCs ranged from good (ICC = 0.66) to excellent (ICC = 0.85). The ICC values above are comparable or even better than those obtained measuring upright static balance with a force platform [44–46], the most employed instrument for static postural balance assessment through the analysis of the CoP trajectory [8]. The reliability of the dynamic tests (both in AP and ML conditions) on the unstable board was corroborated by the low values of SEm and MD_95_ (Tables 5 and 6) and Bland-Altman plots where only a few observations laid just outside the LoA confidence bounds. Moreover, compared to previous studies on balance assessment over wobble board [47], visual examination of the Bland-Altman plots did not show a proportional bias considering the difference and the mean between measures (i.e., y-axis and x-axis, respectively). Compared to previous literature [30], our test allows the upright dynamic postural performance to be investigated separately, considering the AP and ML conditions. Moreover, the proposed three indexes have been validated over the CoP-related parameters instead of a functional test, thus with a more robust methodological approach. While the evaluation of CoP excursions is a common method to measure standing static postural stability objectively [44], the assessment of standing dynamic postural stability is multifaceted, involving more functional than objective tests and considering parameters that are usually specific to the study design (e.g., angle error, recovery step count, surface electromyography) [25]. Although one of these methods (i.e., computerized dynamic posturography) is objective and widely accepted as a reference method [25], it requires expensive and cumbersome instruments. Notably, the assessment presented in the present work and its validation would reduce the lack of standardization and objectiveness among methods of measuring dynamic balance [25]. Moreover, oscillations of an unstable board can be easily measured with an affordable 3D IMU [48], without affecting the portability of the board or without necessarily having to resort to expensive optoelectronic systems or force platforms [31].

Indeed, IMUs have been demonstrated to be valid and accurate devices in kinematics measurements [49], also when compared to motion capture systems [50, 51]. Thus, the device could represent a cost-effective option for measuring dynamic balance similarly to other low-cost systems [9, 52].

Introducing this affordable and objective test for the dynamic balance assessment is essential when considering the rising necessity of evaluating postural dynamic performance. Indeed, the same postural control mechanisms (i.e., cerebral cortex, basal ganglia, cerebellum, brainstem, and spinal cord) have different weights in static and dynamic postural regulations [2]. A quiet stance represents a predictable context where the subject is mainly unaware of the adjustments of postural muscles. Thus, postural regulation mainly occurs at brainstem-spinal levels with local neural loops of assistance [53]. Conversely, in dynamic tasks, a higher involvement of the cognitive process of postural control occurs with a prevalence of supra-spinal postural strategy [2]. Therefore, postural balance assessments should include both static and dynamic assessments [16].

The present study has some limitations. Due to the construction of the unstable board, which allows oscillations only around a single axis, it was not possible to collect the AP and ML oscillations within the same trial. Although TDS includes both AP and ML oscillations, it partially overcomes this issue because it bases on different trials. Another limitation is represented by the radius of curvature of the unstable board that determines the difficulty of the balance task with the same amount of oscillation (i.e., the higher the radius, the easier the maintenance of the balance over the board). Indeed, the radius we adopted surely fit with the population tested but could be too challenging with other populations (e.g., children, elderly, and frail populations). Thus, the tested reliability and validity should be applied cautiously to other populations different from young healthy adults.

In conclusion, the kinematic parameters derived from the unstable board seem valid and reliable indexes for assessing dynamic postural control in physically active young adults. Since these parameters can be measured with an IMU [27, 48], the system could become a valid and economical complement of computerized dynamic posturography to assess dynamic postural control objectively. Moreover, the small size of the system, compared to the other cumbersome and fixed computerized systems available in the market, makes it a valid tool for the on-site dynamic balance assessment. From a practical point of view, we recommend a brief familiarization with the unstable board before performing the balance tests; then, we suggest at least three trials of thirty seconds each. MD_95_ values allow considering, within 95% confidence intervals, if changes in balance parameters (i.e., FB, FiB, and GB) reflect real changes in the dynamic balance performance and not differences that are within what might be reasonably expected given the measurement error of the dynamic balance tests proposed.
